# Sildenafil, a cyclic GMP phosphodiesterase inhibitor, induces microglial modulation after focal ischemia in the neonatal mouse brain

**DOI:** 10.1186/s12974-016-0560-4

**Published:** 2016-04-28

**Authors:** Raffaella Moretti, Pierre-Louis Leger, Valérie C. Besson, Zsolt Csaba, Julien Pansiot, Lorena Di Criscio, Andrea Gentili, Luigi Titomanlio, Philippe Bonnin, Olivier Baud, Christiane Charriaut-Marlangue

**Affiliations:** University Paris Diderot, Sorbonne Paris Cité, INSERM, UMR 1141, 75019 Paris, France; University degli Studi di Udine, Udine, Italy; UPMC-Paris6, AP-HP, Hôpital Armand Trousseau, Réanimation Néonatale et Pédiatrique, 75012 Paris, France; Pharmacologie de la Circulation Cérébrale - EA4475, Faculté des Sciences Pharmaceutiques et Biologiques, University of Paris Descartes, Paris, France; University Paris Diderot, Sorbonne Paris Cité, AP-HP, Hôpital Lariboisière, Physiologie Clinique, Explorations-Fonctionnelles, 75010 Paris, France; University Paris Diderot, Sorbonne Paris Cité, INSERM, U965, 75010 Paris, France; University Paris Diderot, Sorbonne Paris Cité, AP-HP, Hôpital Robert Debré, Urgences Pédiatriques, 75019 Paris, France; University Paris Diderot, Sorbonne Paris Cité, AP-HP, Hôpital Robert Debré, Réanimation Néonatale, 75019 Paris, France; INSERM UMR 1141, Hopital Robert Debré, 48 bd Serurier, 75019 Paris, France

**Keywords:** Neonatal mice, Focal ischemia, Microglia, M2 microglia, Microcirculation

## Abstract

**Background:**

Perinatal ischemic stroke is the most frequent form of cerebral infarction in neonates; however, evidence-based treatments are currently lacking. We have previously demonstrated a beneficial effect of sildenafil citrate, a PDE-5 inhibitor, on stroke lesion size in neonatal rat pups. The present study investigated the effects of sildenafil in a neonatal mouse stroke model on (1) hemodynamic changes and (2) regulation of astrocyte/microglia-mediated neuroinflammation.

**Methods:**

Ischemia was induced in C57Bl/6 mice on postnatal (P) day 9 by permanent middle cerebral artery occlusion (pMCAo), and followed by either PBS or sildenafil intraperitoneal (i.p.) injections. Blood flow (BF) velocities were measured by ultrasound imaging with sequential Doppler recordings and laser speckle contrast imaging. Animals were euthanized, and brain tissues were obtained at 72 h or 8 days after pMCAo. Expression of M1- and M2-like microglia/macrophage markers were analyzed.

**Results:**

Although sildenafil (10 mg/kg) treatment potently increased cGMP concentrations, it did not influence early collateral recruitment nor did it reduce mean infarct volumes 72 h after pMCAo. Nevertheless, it provided a significant dose-dependent reduction of mean lesion extent 8 days after pMCAo. Suggesting a mechanism involving modulation of the inflammatory response, sildenafil significantly decreased microglial density at 72 h and 8 days after pMCAo. Gene expression profiles indicated that sildenafil treatment also modulates M1- (*ptgs2*, *CD32* and *CD86*) and M2-like (*CD206*, *Arg-1* and *Lgals3*) microglia/macrophages in the late phase after pMCAo. Accordingly, the number of COX-2^+^ microglia/macrophages significantly increased in the penumbra at 72 h after pMCAo but was significantly decreased 8 days after ischemia in sildenafil-treated animals.

**Conclusions:**

Our findings argue that anti-inflammatory effects of sildenafil may provide protection against lesion extension in the late phase after pMCAo in neonatal mice. We propose that sildenafil treatment could represent a potential strategy for neonatal ischemic stroke treatment/recovery.

**Electronic supplementary material:**

The online version of this article (doi:10.1186/s12974-016-0560-4) contains supplementary material, which is available to authorized users.

## Background

Perinatal/neonatal arterial stroke is a cerebrovascular event occurring around the time of birth, with pathological or radiological evidence of focal arterial infarction mainly affecting the middle cerebral arterial territory, with an incidence of 1/2800 to 1/5000 live births. There is currently no evidence-based treatment for neonates with stroke [1].

Decreased regional cerebral blood flow (CBF) is the principal factor determining the topography of tissue injury after cerebral ischemia and/or hypoxia-ischemia (HI) in the mature and immature rodents [2]. Partially maintained blood flow in the penumbral tissue after stroke is primarily due to the recruitment of collateral circulation in the brain. Collateral recruitment involves nitric oxide (NO)-dependent vasodilatation, and the effects of endogenous NO may be enhanced by preventing cGMP degradation by phosphodiesterases (PDEs). In particular, sildenafil and tadalafil, two potent selective PDE-5 inhibitors, prolong the action of cGMP in multiple vascular territories [3]. Interestingly, sildenafil treatment, by increasing CBF, reduced HI damage in the P7 rat brain [4]. Sildenafil has been reported to promote neurorestoration in rat models of stroke as measured by neurogenesis, synaptogenesis, and angiogenesis [5] and to reduce proliferation of astrocytes and microglia in chronic inflammatory diseases [6].

While microglia are considered as conductors of the inflammatory response in the central nervous system (CNS), their contribution to the progression of ischemic stroke remains debated [7]. Microglia/macrophages may transition between inflammatory and healing phenotypes in response to specific activation cues (review in [8]). While the M1 or classic phenotype is neuroinflammatory and neurotoxic, the M2 phenotype supports the recovery or neural tissue and is characterized by the production of anti-inflammatory mediators and neurotrophic factors [9]. Although the action of sildenafil on neuroinflammation is not fully understood, the cGMP-PDE inhibitor zaprinast was shown to significantly reduce the inflammatory response of astrocytes and microglia/macrophages in adult rats submitted to focal traumatic brain injury [10].

Revealing molecular mechanisms and pharmacological treatments that promote the transition of microglia/macrophages towards a phenotype that facilitates the repair of the neonatal brain after ischemic damage is of therapeutic interest. The goal of this study is to examine if sildenafil citrate (1) mediates CBF and reduces lesion damage and (2) modifies the modulation of microglia/macrophage between M1 and M2 phenotypes in P9 mice subjected to ischemia after permanent middle cerebral artery occlusion (pMCAo).

## Methods

### Ethics statement

All experiments were carried out (license A75-19-01, French Department of Agriculture) in accordance with the European Committee’s Council Directive and performed to comply with the ethical guidelines of the Robert Debre Hospital Research Council Review Board and have been approved by the local ethic committee (Paris7, France).

### Ischemia

pMCAo was carried out under isoflurane anesthesia in 30 % O_2_ and 70 % N_2_O in P9 C57Bl/6 mouse (Janvier, Le Genest-St. Isle, France; 4.6 ± 0.6 g, *n* = 148). Pups were sacrificed at 72 h and/or 8 days after pMCAo. Two investigators, both blinded to the treatment group, determined the size of the lesion in each animal [11].

### Drug treatment

In the first set of experiments, animals were randomly divided into five groups and treated with either PBS or a single dose of sildenafil citrate (0.5, 2.5, 10, and 15 mg/kg; Pfizer, France), given intraperitoneally (i.p.) 5 min after pMCAo. In the second set of experiments, animals were randomly divided into three groups and treated with either PBS or a single dose of sildenafil citrate (0.5 and 10 mg/kg, i.p.) 5 min after pMCAo (see Additional file 1: Figure S1 for an outline of the experimental procedure).

### cGMP measurement

Competitive enzyme immunoassay (Cayman Chemical Company, Ann Arbor, MI, USA) was used to quantify cGMP in the forebrain, according to the manufacturer’s instructions. Whole brains at P9 were harvested 1 and 3 h after the administration of sildenafil (0.5 and/or 10 mg/kg) and immediately frozen at −80 °C until measurements were performed.

### Ultrasonographic brain imaging

Thermoregulated mice (*n* = 6 per group) were subjected to ultrasound measurements under inhaled isoflurane anesthesia (0.8 % in air via a facemask) using an echograph (ACUSON S3000, Siemens, Erlangen, Germany) equipped with a 14.5-MHz linear transducer (14L5 SP) [12]. Heart rate and time-average mean blood flow velocities (mBFVs) were measured in both intracranial carotid arteries (ICA) and the basilar trunk (BT) at baseline and 1 h after pMCAo and PBS and sildenafil (10 mg/kg) treatment.

### Laser speckle contrast (LSC) imaging

Imaging was carried out using a full-field laser perfusion imager (FLPI2, Moor Instruments Ltd., Axminster, UK) [12]. Briefly, thermoregulated and anesthetized (with 1 % isoflurane inhalation in air via a facemask) mouse pups (*n* = 6 per group) were placed in lateral decubitus and the skin incised to provide access to the skull. Vegetable oil was applied to avoid surface dryness. Speckle images (760 × 568 pixels) were collected at 0.25 Hz (4 ms exposure time) and recorded at baseline, after MCA electrocoagulation and 5, 15, 60, and 90 min after treatment. Blood fluxes were measured in three cortical regions of interest (ROI, 50 × 50 pixels), ROI 1 present in the core of the infarct and ROI 2 and ROI 3 in the proximal and distal penumbra, respectively. Fluxes in the ROIs were expressed in arbitrary units using a 16-color palette.

### Real-time quantitative reverse transcriptase polymerase chain reaction (qRT-PCR)

Animals were sacrificed at 72 h and 8 days after pMCAo (*n* = 8 per group). The harvested tissues correspond to the parietal cortex, including the ischemic core (pale zone) and penumbra at 72 h, and the penumbra at 8 days as the pale zone being a cavity. Total RNA was extracted using RNeasy lipid tissue mini kit (Qiagen, Courtaboeuf, France). RNA purity and quality were assessed by spectrophotometry with the NanoDrop™ apparatus (Thermo Scientific, Wilmington, DE, USA). Total RNA (1–2 μg) was subjected to reverse transcription using the iScript™ cDNA synthesis kit (Bio-Rad, Marnes-la-Coquette, France). RT-qPCR was performed in duplicates for each sample using SYBR Green Supermix (Bio-Rad) for 40 cycles with a 2-step program (5 s of denaturation at 96 °C and 10 s of annealing at 60 °C). Amplification specificity was assessed by melting curve analysis. Primers were designed using Primer3 software. Sequences and NCBI references are provided in Additional file 2: Table S1. Abundance of transcripts of interest was expressed relative to the expression of the reference gene, glyceraldehyde 3-phosphate dehydrogenase (*GAPDH*). Analyses were performed with the Bio-Rad CFX Manager 2.1 software.

### Immunohistochemistry

Coronal 16-μm thick paraffin sections at the MCA and hippocampal level (corresponding to 3.27 and 4.83 mm—atlas of the developing mouse brain at P6) were stained for mouse anti-GFAP (Sigma-Aldrich G3893; 1:500 dilution), goat anti-Iba1 (Abcam ab5076; 1:500), rabbit anti-Iba1 (Wako, 019-19741; 1:1000), goat anti-MRC1 (MMR/CD206 polyclonal Goat IgG, R&D system, AF2535; 1:400), rabbit anti-COX-2 (Abcam; 1:200), goat anti-arginase-1 (Santa Cruz Biotechnology, Inc., sc-18354; 1:200), rabbit anti-Glut1 (54-KDa form, Millipore, Molsheim, France; 1:700), and tomato lectin (Vector Laboratories, 1:500). For immunofluorescence, secondary antibodies coupled with the green marker Fluoroprobe S488 (Interchim, Montluçon, France) or the red fluorescent marker cyanine 3 (Jackson Immuno Research laboratories, West grove, PA) were used. For confocal imaging, sections were analyzed using a Leica TCS SP8 confocal scanning system (Leica Microsystems, Wetzlar, Germany) equipped with 405-nm diode, 488-nm argon, and 561-nm DPSS lasers. A series of optical sections separated by 0.3 μm was collected using the ×63 HC PL APO CS2 objective (numerical aperture 1.40). For each optical section, double- or triple-immunofluorescence images were acquired in sequential mode to avoid potential contamination by fluorescence emission overlap. The orthogonal sectioning and 3D viewing were produced by the Leica Application Suite X-software (Leica Microsystems). Images were equally adjusted for brightness and contrast, and composite illustrations were built in Adobe Photoshop CS3 (Adobe Systems, San Jose, CA).

### Cell quantification

GFAP and Iba-1-positive cells were counted in a blind manner in the penumbra region on three coronal sections for animal at 72 h and 8 days after ischemia and in age-matched controls using a ×20 objective. Iba-1-Arg-1 and Iba1-COX-2 double-positive cells were counted in the same regions with a ×20 lens and then merged images were created with Fiji software (http://fiji.sc/Fiji).

### Statistical analysis

Values are expressed as mean ± SD (BF measures and lesion volume) and mean ± SEM (RT-qPCR, immunohistochemistry, and immunofluorescence). BFV and CBF values measured in the different ROIs were compared using repeated measures ANOVA and a post hoc Newman-Keuls test to analyze differences between the two groups. One-way ANOVA followed by a Newman-Keuls post hoc test was used for the difference between two groups (RT-qPCR, immuno-labeled cells, and lesion volumes). Statistical analyses were performed using GraphPad PRISM version 5.0 (GraphPad Software, San Diego, CA).

## Results

### Setup of the animal model and effect of sildenafil on CBF and lesion size

**Fig. 1:**
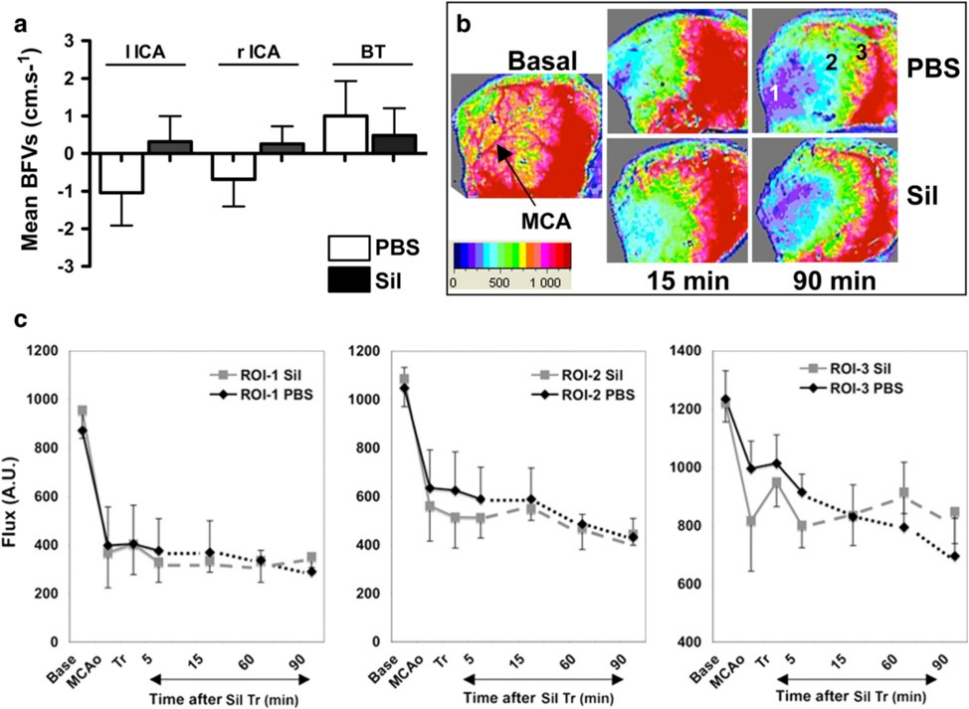
Redistribution of blood flow after permanent MCAo (pMCAo) in C57BL/6 P9 mice using ultrasound Doppler and laser speckle contrast (LSC) imaging. **a** Variation in mean blood flow velocity (mBFV) in the large cerebral arteries (*lICA* left ICA, *rICA* right ICA, *BT* basilar trunk) 1 h after pMCAo and administration of PBS and/or sildenafil (10 mg/kg; *n* = 6 per group). **b, c** Representative color-coded LSC images **(b)** and time-course of cortical blood flow **(c)** taken in basal, upon MCAo, shortly after sildenafil treatment (*Tr*, *PBS*, and/or sildenafil, *Sil*, 10 mg/kg), and after 5, 15, 60, and 90 min in three ROIs (ROI 1, core; ROI 2 and ROI 3 close and far away from the penumbra, respectively). BF is expressed in arbitrary units (A.U.) according to the color-coded scale (0 to 1500)

In the first set of experiments (Additional file 1: Figure S1), animals were assessed by color-coded pulsed Doppler ultrasound imaging at baseline and after pMCAo. As compared to basal mBFV, pMCAo induced a significant transient decrease in mBFV in the left ICA and a consecutive significant increase in mBFV in the basilar trunk (BT) 15 min after pMCAo (Additional file 1: Figure S2A). One hour after pMCAo, no significant change in mBFV was observed in the three large arteries (data not shown). One hour after pMCAo and sildenafil administration (10 mg/kg, *n* = 8 each) mBFVs were not modified in the three large arteries as compared to a single PBS administration (Fig. 1a). Heart rates in the sildenafil-treated animals (395 ± 67 bpm) were not significantly different from PBS-treated animals (428 ± 66 bpm) at this time point. No difference was observed between the different doses of sildenafil on mBFVs measured in the large arteries (data not shown), except for the 15 mg/kg dose (Additional file 1: Figure S2B).

Representative patterns and values of cortical BF using LSC imaging are shown in Fig. 1b, c. Immediately after pMCAo, BF dropped in the ROI 1 (core) to a mean value around 40–42 % of basal BF in both groups of animals (*n* = 6 each) and was reduced further to 33–36 % of basal at 90 min after pMCAo. A similar drop in BF was measured in the ROI 2 and in a less severe manner in the ROI 3. BF was further reduced at 90 min after pMCAo, in particular in ROI 3. As expected, sildenafil treatment (10 mg/kg) significantly increased the concentration of intracellular cGMP to 1.91 ± 0.38 and 1.87 ± 0.31 pmol/mL at 1 and 3 h after administration, respectively, as compared to PBS-treated animals (0.08 ± 0.02 pmol/mL, *p* < 0.001) (Additional file 1: Figure S3). No statistical difference in cGMP concentration was found between the different doses used. Nevertheless, sildenafil treatment did not significantly modify the BF profile in the three distinct ROIs at any time point.

**Fig. 2:**
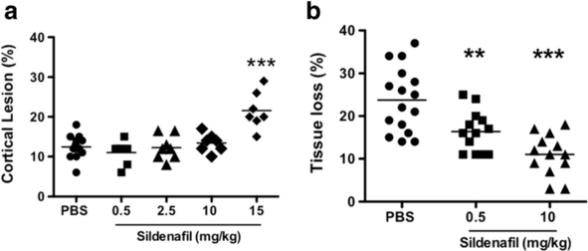
Effect of sildenafil citrate on the cortical infarct volumes and tissue loss measured 72 h and 8 days after pMCAo, respectively. Sildenafil citrate was given after pMCAo by a single intraperitoneal injection either with PBS or 0.5, 2.5, 10, and 15 mg/kg sildenafil. **a** Cortical lesion at 72 h. **b** Tissue loss at 8 days. *Horizontal bar* represents the mean. Note that the 15 mg/kg dose is toxic by increasing the infarct volume. Each *dot* represents one animal. Data were assessed via an ANOVA, and when significant the results of the Newman-Keuls post-test are shown, ** *p* < 0.01, *** *p* < 0.001 sildenafil dose vs PBS

Mean cortical infarct volume at 72 h after pMCAo was 12.5 ± 3.0 % (*n* = 14, one animal died) in PBS-treated animals. Sildenafil at the three lower doses tested (0.5, 2.5, and 10 mg/kg) did not change the mean infarct volume (11.0 ± 3.0 %, *n* = 7; 12.2 ± 3.1 %, *n* = 8; and 13.4 ± 2.3 %, *n* = 7, respectively, N.S.), whereas it increased both infarct volume (21.6 ± 4.6 %, *n* = 7, *p* < 0.001, Fig. 2a) and mortality (3/10 animals died) at 15 mg/kg, suggesting toxicity at high doses (Fig. 2a). Therefore, the 15-mg/kg dose was not further used in the following experiments. Eight days after pMCAo, lesion volume significantly increased up to 23.8 ± 7.5 % (*n* = 16, *p* < 0.01 vs 72 h) in PBS-treated animals and became cystic. Sildenafil significantly counteracted this increase in a dose-dependent manner to 16.4 ± 4.8 % (sildenafil 0.5 mg/kg; *n* = 13; *p* < 0.01) and 11.0 ± 4.8 % (sildenafil 10 mg/kg; *n* = 13; *p* < 0.001) (Fig. 2b).

### Effect of sildenafil treatment on glial activation

Reactive astrogliosis manifested by a significant increase in GFAP immunoreactivity was clearly observed around the lesion in PBS-treated animals at 72 h after pMCAo (Additional file 1: Figure S4 and Additional file 1: Figure S5A, B). Surprisingly, sildenafil significantly increased the prevalence of GFAP^+^ cells at 10 mg/kg. Conversely, sildenafil significantly reduced the number of GFAP^+^ cells 8 days after pMCAo as compared to PBS-treated animals. No significant difference was observed between PBS and 0.5 mg/kg sildenafil treatment at either time point (Additional file 1: Figure S4).

**Fig. 3:**
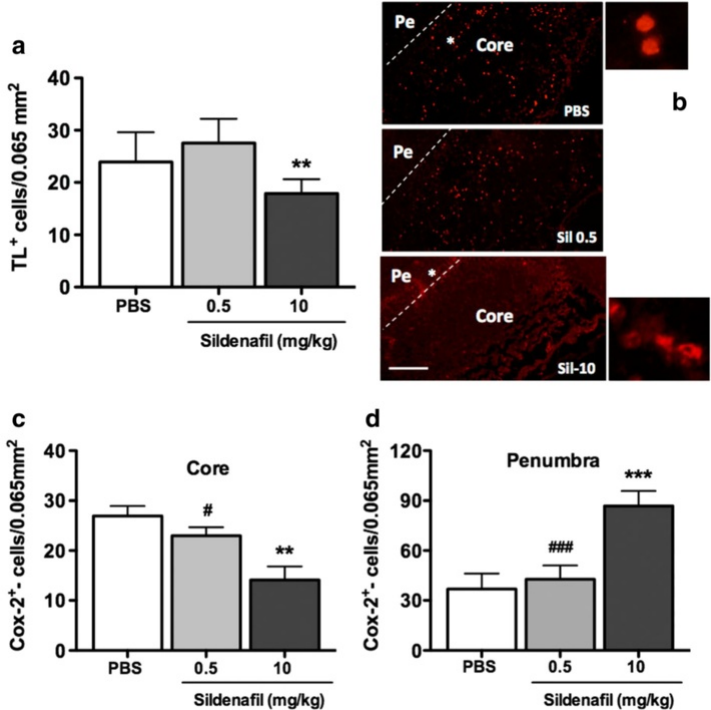
Effect of sildenafil citrate on the density of microglia/macrophages cells and COX-2 expression at 72 h after pMCAo. **a** Quantitative analysis of tomato-lectin (*TL*, *n* = 6–7 per group) in PBS, Sil-0.5, and Sil-10 (mg/kg)-treated animals. **b–d** Localization and quantification of COX-2^+^ cells in the core and/or the penumbra in PBS- and sildenafil-treated animals. ** *p* < 0.01, ***p < 0.001, sildenafil vs PBS treatment. ^##^ *p* < 0.01, ^###^ *p* < 0.001, sildenafil 0.5 vs sildenafil 10. Scale bar represents 100 μm **(b)** and 50 μm (*enlarged panels*)

A high abundance of microglia/macrophages (stained by tomato lectin (TL)) was detected in the penumbra (including the white matter) at 72 h. Sildenafil treatment significantly decreased the number of TL^+^-cells at 10 but not 0.5 mg/kg (Fig. 3a and Additional file 1: Figure S5C, D). At this time point, cells positive for the M1-like marker COX-2^+^ [13] were found in the ischemic core in PBS-treated animals, whereas they were mostly observed in the penumbra in 10 mg/kg (but not 0.5 mg/kg) sildenafil-treated animals (Fig. 3b–d). In contrast, 8 days after pMCAo the number of microglia/macrophages stained by Iba-1 were significantly reduced by sildenafil treatment (0.5 and/or 10 mg/kg dose) (Fig. 6b).

**Fig. 4:**
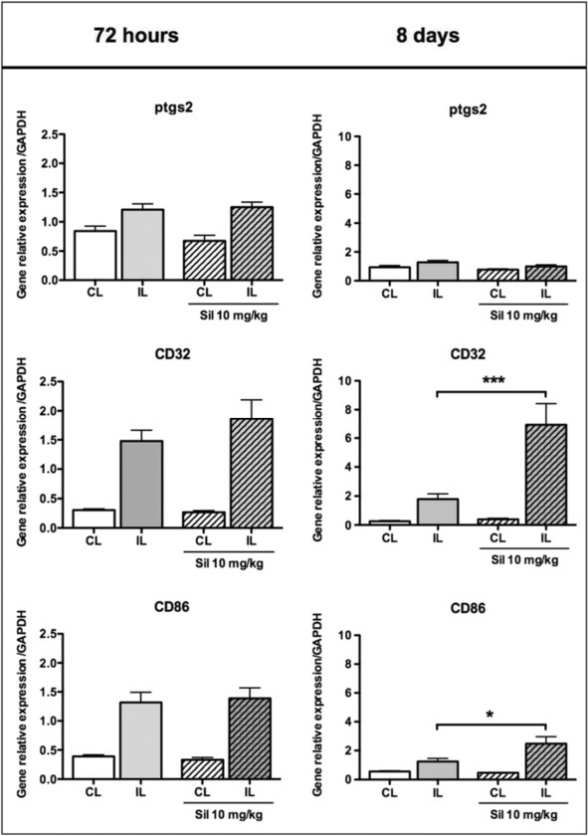
Gene expression of microglia/macrophage M1-like markers in PBS- and sildenafil (*Sil*)-treated animals, 72 h and 8 days after pMCAo. M1 markers were measured in the ipsilateral (*IL*) and contralateral (*CL*) sides in PBS (*plain bars*) and sil-10 (*hatched bars*) animals. Data are mean ± SEM. Data were assessed via an ANOVA, and when significant, the results of the Newman-Keuls post-test are shown; *p < 0.05, and ***p < 0.001, compared to PBS

**Fig. 5:**
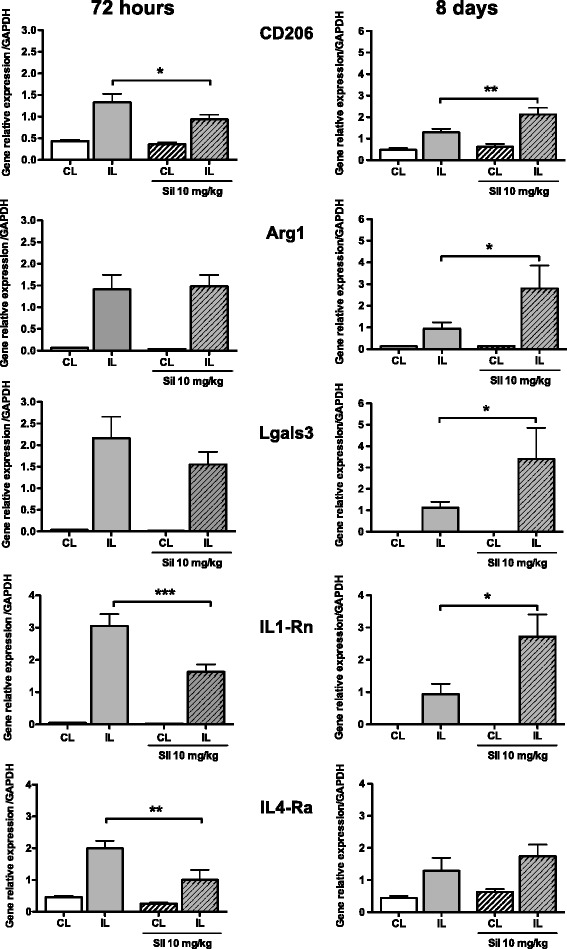
Gene expression of microglia/macrophage M2-like markers in PBS- and sildenafil (*Sil*)-treated animals, 72 h and 8 days after pMCAo. M2 markers were measured in the ipsilateral (*IL*) and contralateral (*CL*) side in PBS- (*plain bars*) and sil-10 (*hatched bars*) animals. Data are mean ± SEM. Data were assessed via an ANOVA, and when significant, the results of the Newman-Keuls post-test are shown; *p < 0.05, **p < 0.01, and ***p < 0.001, compared to PBS

**Fig. 6:**
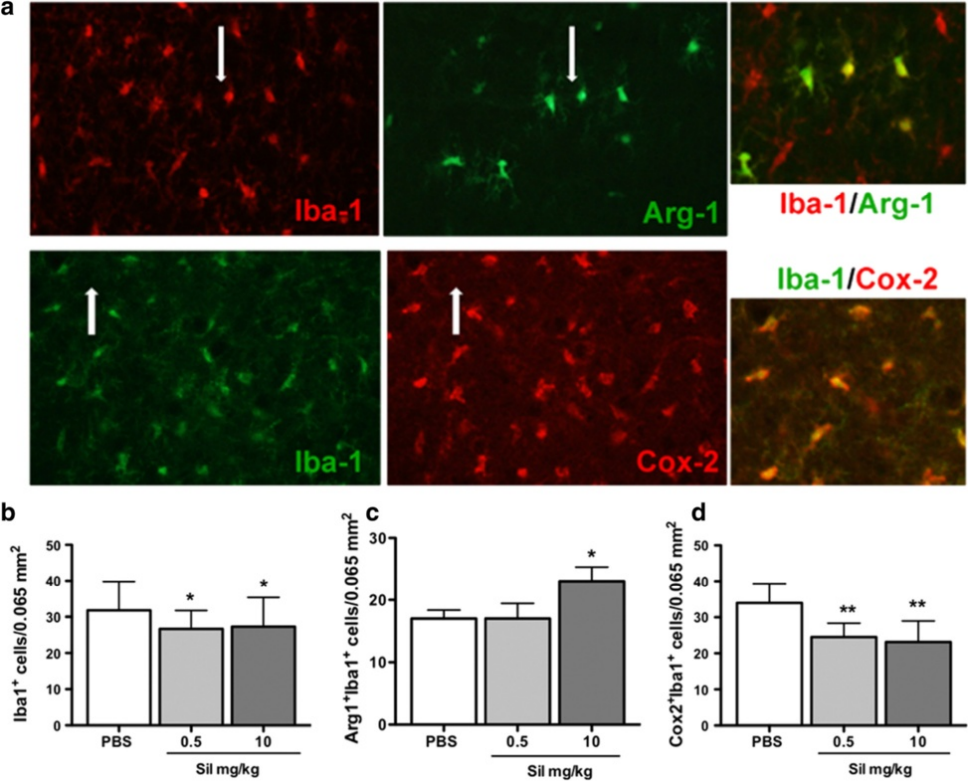
Effect of sildenafil on the number of Iba-1-positive microglia and M2 and M1 phenotypes 8 days after pMCAo. **a** Typical example of double fluorescence labeling in the cortical penumbra for Iba-1 and Arg-1 and Iba-1 and COX-2 cells in a PBS-treated animal. Note that almost Iba-1^+^ cells are co-stained with COX-2 marker (*white arrows*), which is not the case for double-stained Iba-1^+^-Arg-1^+^ cells. **b–d** Quantification of the number of Iba-1^+^, Iba-1^+^-COX-2^+^, and Iba-1^+^-Arg-1^+^ microglia cells in PBS-treated and sildenafil-treated (0.5 and 10 mg/kg) animals. Data are mean ± SEM. Data were assessed via an ANOVA, and when significant, the results of the Newman-Keuls post-test are shown. *p < 0.05, **p < 0.01, sildenafil vs PBS treatment

**Fig. 7:**
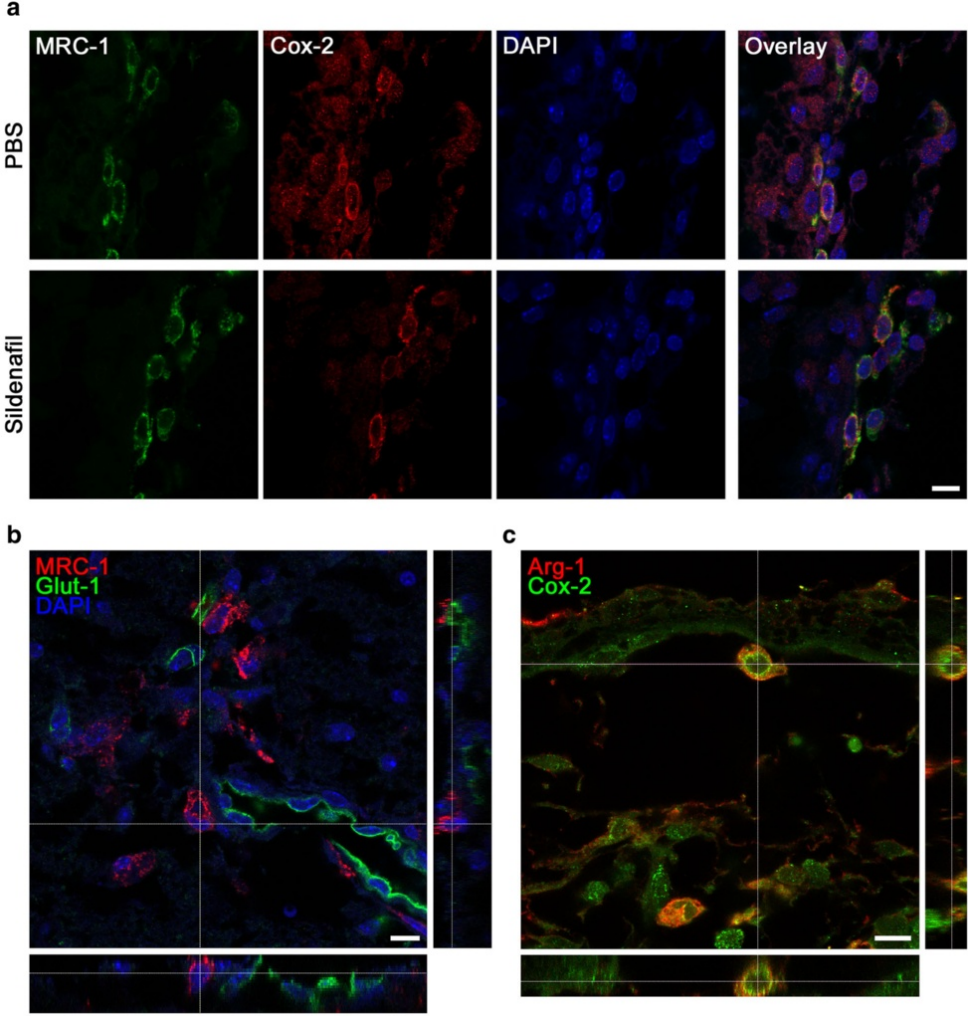
3D confocal analysis of double-labeled microglia and vessels after pMCAo and PBS and/or sildenafil (0.5 mg/kg) treatment. **a** Co-localization of M1 (COX-2 in *red*) and M2 (MRC-1 in *green*) markers in the leptomeningeal membranes, 72 h after ischemia. **b** MRC-1 (in *red*) is present in the perivascular microglia (in *green*). **c** Co-localization of M1 (COX-2 in *green*) and M2 (Arg-1 in *red*) markers in the leptomeningeal membranes 8 days after pMCAo, in a PBS-treated animal. Scale bar represents 10 μm **(a–c)**

We then focused on the phenotype of microglial cells at both transcriptional (Figs. 4 and 5) and translational (Figs. 6 and 7) levels in the cortical tissue obtained from PBS- and sildenafil-treated animals. Seventy-two hours after pMCAo and PBS treatments, most of the markers (M1- and M2-like) were increased with the exception of ptgs2 (COX-2). As compared to PBS, sildenafil (10 mg/kg) did not change the expression of M1-like (*ptgs2*, *CD32* and *CD86*) markers 72 h after ischemia. In contrast, a significant increase was measured for *CD32* and *CD86* 8 days after pMCAo (Fig. 4). At 72 h after pMCAo, sildenafil treatment (10 mg/kg) significantly decreased mRNA expression of M2-like markers (*CD206*, *IL1-Rn*, and *IL4-Ra*) but not *Arg-1* and *Lgals3*. In contrast, most of these markers (*CD206*, *Arg-1*, *Lgals3* and *IL1-Rn*) were increased 8 days after pMCAo (Fig. 5).

To further address the effect of sildenafil treatment on M1 and M2 markers, we examined the co-localization of Iba-1^+^ (a marker for both M1 and M2 microglia/macrophages) with either the M2-like marker Arg-1 (Fig. 6a, upper panel) or the M1-like marker COX-2 (Fig. 6a, lower panel) in the cortical penumbra. While a global decrease of Iba-1^+^ microglia/macrophage was observed by sildenafil irrespective of dosage (Fig. 6b), an increase of Iba-1^+^Arg-1^+^ double-positive M2 microglia/macrophage was observed at 10 but not 0.5 mg/kg (Fig. 6c), suggesting that a high dosage of sildenafil was required for microglia/macrophages to obtain the M2 status. Interestingly, sildenafil decreased the number of Iba-1^+^COX-2^+^ double-positive M1 microglia/macrophages irrespective of dosage (Fig. 6d), suggesting it does not only affect phenotype polarization and that a lower dose threshold is required for inhibition of neurotoxic M1-type than for activation of neuroprotective M2-type microglia/macrophages.

### Co-expression of M1- and M2-like markers in perivascular microglia/macrophages

The macrophage mannose receptor MRC-1 (CD206), a marker for M2-type microglia/macrophages, was mainly detected in the leptomeninges from where they entered the neocortex along the penetrating arterioles in the penumbral tissue at 72 h (Fig. 7) and 8 days after pMCAo (data not shown). On confocal images, we observed the co-localization of MRC-1 with the M1 marker COX-2 (Fig. 7a) indicating either a mixed phenotype or lack of specificity of this marker. MRC-1 expression was present in macrophages/microglia localized around microvessels labeled with the endothelial marker Glut-1 (stained with 54 kDa Glut-1 protein, which regulates import of glucose from the blood to the brain across the endothelial cells of the blood-brain barrier) and present in the penumbra (Fig. 7b and Additional file 1: Figure S6). A few COX-2^+^/Arg-1^+^-cells (2–4 cells per brain section) were still detected in the leptomeninges in both PBS- (Fig. 7c) and sildenafil- (10 mg/kg, data not shown) treated animals 8 days after pMCAo.

## Discussion

In this study, we show that brain lesions induced by pMCAo in P9 mouse pups evolve between 72 h and 8 days post-ischemia and that the selective inhibition of PDE-5 by sildenafil dose-dependently reduces lesion extent at 8 days post-pMCAo. The protective action of sildenafil appeared to involve a reduction in the overall number of microglia/macrophages in the late phase of lesion development as well as their polarization towards a neuroprotective M2 phenotype.

Whereas lesion volumes decrease after pMCAo in adult mice between 24 h and 7 days [14], they increased between 3 and 8 days in neonatal mice, as previously shown in neonatal rat [15]. Whether this difference reflects upon continued tissue destruction even in late phases after occlusion or reduced growth potential of the neonatal brain is yet unclear. Sildenafil treatment reduced lesion size at 8 days post-pMCAO, similar to that found at 72 h, suggesting that sildenafil prevents lesion enlargement. We previously reported that sildenafil treatment, associated with an early significant increase in cerebral blood flow (CBF), reduces hypoxic-ischemic damage in the P7 rat brain [4]. By using both US and LSC imaging, we were here unable to observe early hemodynamic changes in either sildenafil- or PBS-treated mice. A high variability in the number of leptomeningeal and/or pial vessels may explain the hemodynamic differences observed between the rat and mouse. Indeed, all ischemic models in the rat combine transient occlusion of two arteries at the same time, either pMCAo with transient occlusion of one or both common carotid arteries [2, 16] or blockade of the past external carotid artery-internal carotid artery bifurcation [17, 18]. In contrast, a single pMCAo is sufficient to create ischemic lesions in mice [19]. Together, this suggests that combined occlusion of carotid and MC arteries is necessary to drop the BF in the ipsilateral hemisphere sufficiently for anastomoses to no longer be efficient despite the circle of Willis and to create a lesion in the rat. As BF was only measured up to 90 min after pMCAo, we cannot exclude the contribution of late collateral opening and patency. Together, our data suggest that sildenafil, although given shortly after pMCAo, may have other neuroprotective effects that may develop at later time points of recovery.

The impact of inflammation on post-injury outcome in the developing brain is increasingly recognized. In our pMCAo model in the P9 mouse, strong astrocytic and microglial responses were detected 72 h after injury and persisted for more than 1 week (personal data). We show that sildenafil potentiates reactive astrogliosis 72 h after pMCAo, in agreement with a previous report following focal cryolesions onto the cortex in adult rats treated with zaprinast and killed 3 days thereafter [9]. Zaprinast was expected to accelerate the formation of the glial scar and the regeneration of the injured tissue [10]. Sildenafil similarly increased GFAP immunoreactivity in adult mice submitted to a cortical cryolesions [20]. After brain injury, NO-dependent cGMP formation can occur in the astrocytes following NO synthase-2 induction in activated astrocytes and infiltrating macrophages [21, 22]. Natriuretic peptides (NPs) comprising atrial (ANP), brain NP (BNP), and C-type (CNP) share the same intracellular signal transduction pathways with cGMP/cGMP-dependent protein kinase (cGK) as well as the NO pathway. The NPs/NO/cGMP/cGK pathway has been reported to increase BF and to be critical for neovascularization in vivo [23]. Interestingly, the content of ANP has been reported to increase in reactive astrocytes 3 days after injury in the white matter (WM) from autopsied human brain specimens after brain infarction and upregulation of the ANP/cGMP pathway (ANP is a vasorelaxant) may contribute to an increase in BF in response to the BF reduction in the infarcted area [24]. The increase of GFAP^+^ cells observed here 3 days after pMCAo might be related to an increase in ANP and subsequent BF that we did not evaluate and could further explain the delayed reduction in astrogliosis observed at 8 days after pMCAo.

Another important finding of this study is that sildenafil reduces the recruitment and activation of microglia/macrophages in the penumbral tissue at both 72 h and 8 days after pMCAo, as found for zaprinast in the cortical cryolesion model [9]. Microglial activation may be beneficial or harmful depending on polarization towards a pro-inflammatory M1 or an anti-inflammatory and pro-healing M2 phenotype [25]. Information regarding the expression of M1 and M2 markers and their temporal and spatial evolution in the ischemic brain are largely lacking. Changes in mRNA expression of M1 and M2 markers were maximally increased between 3–5 and 14 days after transient MCAo in the adult C57BL/6 mice [26].

In our neonatal mouse model, we observed an impact of sildenafil treatment on macrophage/microglia activation and the expression of M1- and M2-like markers. Whereas sildenafil only increased M1-like genes 8 days after ischemia, it decreased M2-like genes expression at the same time point. Cyclooxygenases (COX-1 and COX-2), which produce vasodilatory prostaglandins [27], contribute to neurovascular coupling [28] and could contribute to vasodilation in the penumbra and a subsequent absence of lesion extension in animals treated with sildenafil. Furthermore, cerebrovascular dilations to hypercapnia were demonstrated to be prostanoid dependent and nitric oxide independent in the newborn pig brain [29, 30]. The combined increase of potential vasodilatory mediators in astrocytes and COX-2^+^ cells in the penumbra at 72 h could, therefore, induce patency and/or recruitment of collaterals leading to a reduced damage 5 days after (at P17).

A significant upregulation of M2-like mRNA was detected after sildenafil treatment. In the adult rodent, M1 markers (*iNOS*, *CD11b*, *CD16*, and *CD32*) were still elevated at 14 days after stroke, while M2 markers (*CD206*, *Arg-1*, *Ym1/2* and *IL-10*) were decreased at 7 days after ischemia [31]. The M2 phenotype has a stronger capacity to elicit phagocytosis of dead neurons to avoid secondary inflammatory response and promote tissue regeneration, probably by the ANP/cGMP/cKG pathway [32]. We suggest a shift in polarization towards the M2 phenotype may contribute to the ability of sildenafil to prevent the late-stage extension of lesions in our model. We also found MRC-1 (CD-206) protein in infiltrating macrophages (in the leptomeninges), as previously reported after hypoxia-ischemia in the P7 rat [33]; however, we further demonstrate that MRC-1^+^/CD206 perivascular microglia/macrophages entered the brain via penetrating microvessels. In contrast CD-206^+^ cells were exclusively located in the ischemic core in adult mice 24 h after pMCAo [34]. Interestingly, mixed transitional (Mtran) phenotype has been introduced to characterize double-labeled M1-M2 macrophage/microglia following traumatic brain injury [35]. Such MRC-1^+^/COX-2^+^ and Arg-1^+^/COX-2^+^ macrophage/microglia in the leptomeninges may also represent a transitional Mtran phenotype in our ischemic model.

## Conclusions

In conclusion, our study suggests that sildenafil may prevent the progression of the ischemic lesions in neonatal mice through the modulation of inflammatory pathways including microglial/macrophage polarization. Further investigation is needed to elucidate mechanisms that govern the change in microglia/macrophage phenotype and the regulation of lesion size in the neonatal rodent brain. As the use of sildenafil is indicated for idiopathic pulmonary hypertension [36], it could be explored for immunomodulatory stroke treatment in children.

## Additional files

Additional file 1: Figures S1–S6. Figure S1. Outline of the experimental procedure in P9 C57Bl/6 mice subjected to pMCAo. **Figure S2.** A: mean blood flow velocities (BFVs) in the left and right ICA (lICA, rICA) and basilar trunk (BT) in basal conditions and 15 min after pMCAo. *, #*p* < 0.05 vs basal for each artery. B Variation in arterial blood flow (BF) in P9 mice subjected to pMCAo and administered with PBS (white bars) and/or sildenafil (15 mg/kg, black bars). **Figure S3.** cGMP dosage in the ipsilateral hemisphere in the control (PBS-treated naive mice, *n* = 5) and in sildenafil- (10 mg/kg) treated mice, at 1 and 3 h (h) after treatment. ****p* < 0.001 vs PBS. **Figure S4.** Quantification of GFAP-positive cells in the penumbra at 72 h and 8 days after pMCAo in PBS- and sildenafil-treated animals (*n* = 8 per group). **Figure S5.** Immunohistochemistry for astrocyte (GFAP; A, B) and macrophage (tomato lectin (TL) C–D)/microglia (Iba-1; E, F) in PBS-treated animals (A, C, and E) and sildenafil 10 mg/kg (B, D, and F) at 72 h (A–D) and 8 days (E, F) after pMCAo in mouse pups. **Figure S6.** 3D confocal reconstruction of a microvessel stained with the 54 kDa Glut-1 protein (green) and microglia/macrophage stained with MRC-1 protein (M2-like marker). (PDF 1359 kb) Additional file 2: Table S1. Primer sequences, protein targets, and NCBI references. (PDF 306 kb)
