# Supplementary material for: Sildenafil, a cyclic GMP phosphodiesterase inhibitor, induces microglial modulation after focal ischemia in the neonatal mouse brain
Source: J Neuroinflammation. 2016 Apr 28;13:95. doi: 10.1186/s12974-016-0560-4 (PMC4850658; doi:10.1186/s12974-016-0560-4)
Supplement: Additional file 1: Figures S1–S6. Figure S1. — Outline of the experimental procedure in P9 C57Bl/6 mice subjected to pMCAo. Figure S2. A: mean blood flow velocities (BFVs) in the left and right ICA (lICA, rICA) and basilar trunk (BT) in basal conditions and 15 min after pMCAo. *, #p < 0.05 vs basal for each artery. B Variation in arterial blood flow (BF) in P9 mice subjected to pMCAo and administered with PBS (white bars) and/or sildenafil (15 mg/kg, black bars). Figure S3. cGMP dosage in the ipsilateral hemisphere in the control (PBS-treated naive mice, n = 5) and in sildenafil- (10 mg/kg) treated mice, at 1 and 3 h (h) after treatment. ***p < 0.001 vs PBS. Figure S4. Quantification of GFAP-positive cells in the penumbra at 72 h and 8 days after pMCAo in PBS- and sildenafil-treated animals (n = 8 per group). Figure S5. Immunohistochemistry for astrocyte (GFAP; A, B) and macrophage (tomato lectin (TL) C–D)/microglia (Iba-1; E, F) in PBS-treated animals (A, C, and E) and sildenafil 10 mg/kg (B, D, and F) at 72 h (A–D) and 8 days (E, F) after pMCAo in mouse pups. Figure S6. 3D confocal reconstruction of a microvessel stained with the 54 kDa Glut-1 protein (green) and microglia/macrophage stained with MRC-1 protein (M2-like marker). (PDF 1359 kb) [file 12974_2016_560_MOESM1_ESM.pdf]

Supplemental content – **Moretti et al.**

Sildenafil, a cyclic GMP phosphodiesterase inhibitor, induces microglial modulation after focal ischemia in the neonatal mouse brain

Supplemental results and Figures

The experiments done in this study were performed in two sets, one with an end-point at 72 hours and the second with an endpoint at 8 days after pMCAo.

Supplemental Figure 1

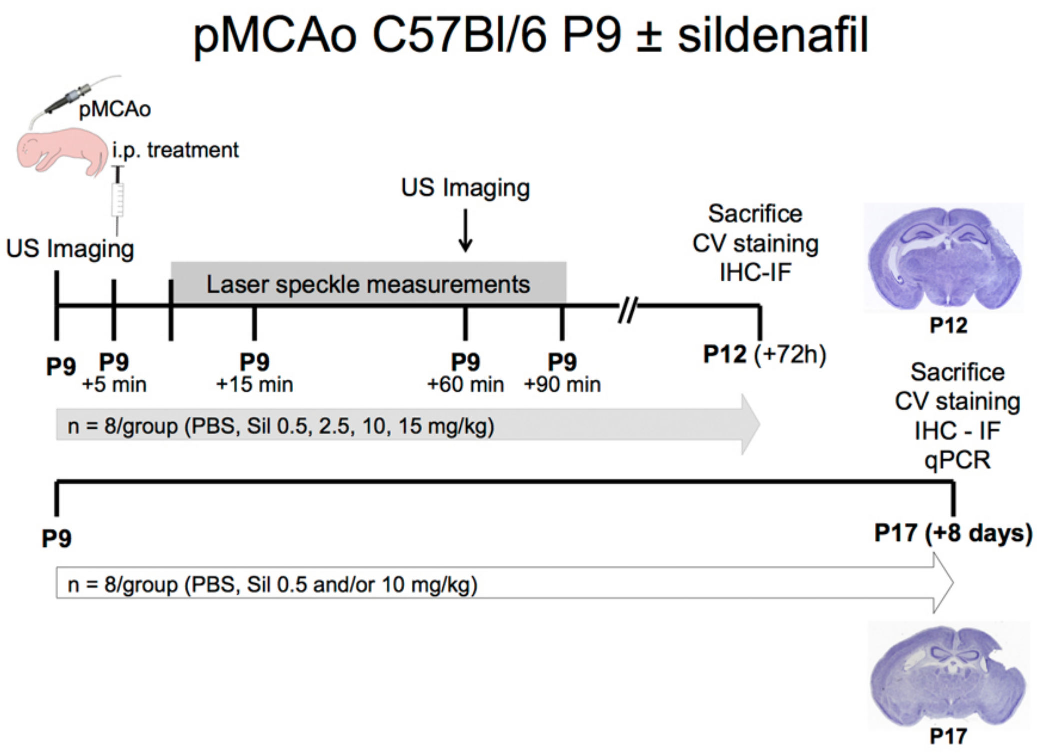

Figure 1: Outline of the experimental procedure in P9 C57Bl/6 mice subjected to pMCAo. CV= cresyl violet, IHC = immunohistochemistry, IF = immunofluorescence. Note that a cavity is observed 8 days after pMCAo (corresponding to the core of the lesion).

Supplemental Figure 2

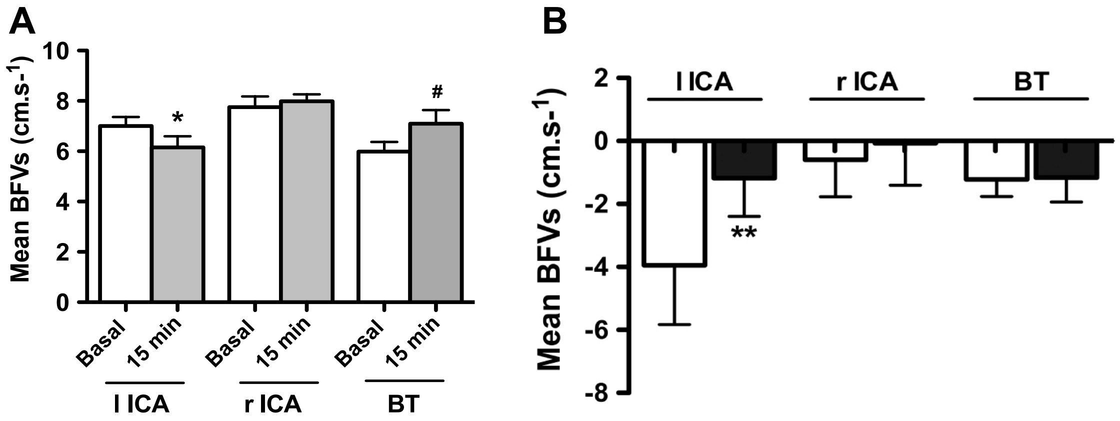

Figure 2: **A**: Mean blood-flow velocities (BFVs) in the left and right ICA (lICA, rICA) and basilar trunk (BT) in basal conditions and 15 min after pMCAo. $^{*,\#}$*p*<0.05 vs Basal for each artery. **B**: Variation in arterial blood flow (BF) in P9 mice subjected to pMCAo, and administered with PBS (white bars) and/or sildenafil (15 mg/kg, black bars). Note that sildenafil, at this dose, reduced low BF in the left ICA (lICA). However, P9 mice have significant increased heart rates compared to PBS (from 429±30 to 477±44 bpm, p<0.05). This increased cardiac function is suggested to be deleterious because of the increasing number of died animals (n=3).

Supplemental Figure 3

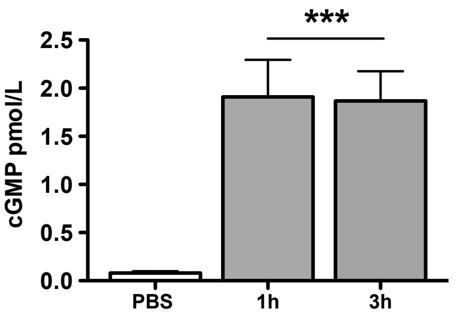

Figure 3: cGMP dosage in the ipsilateral hemisphere in control (PBS-treated naive mice, n=5), and in sildenafil- (10 mg/kg) treated mice, at 1 and 3 hours (h) after treatment. ***p<0.001 *vs* PBS.

Supplemental Figure 4

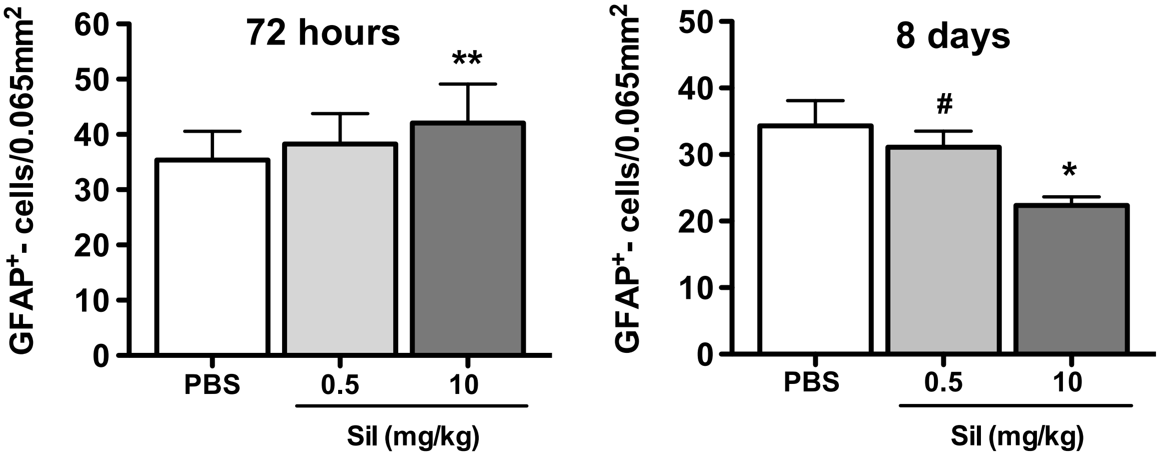

Figure 4: Quantification of GFAP-positive cells in the penumbra at 72 hours and 8 days after pMACo in PBS- and Sildenafil-treated animals (n=8 per group). *p<0.05, **p<0.001 *vs* PBS. #p<0.05 sildenafil 0.5 *vs* sildenafil 10.

Supplemental Figure 5

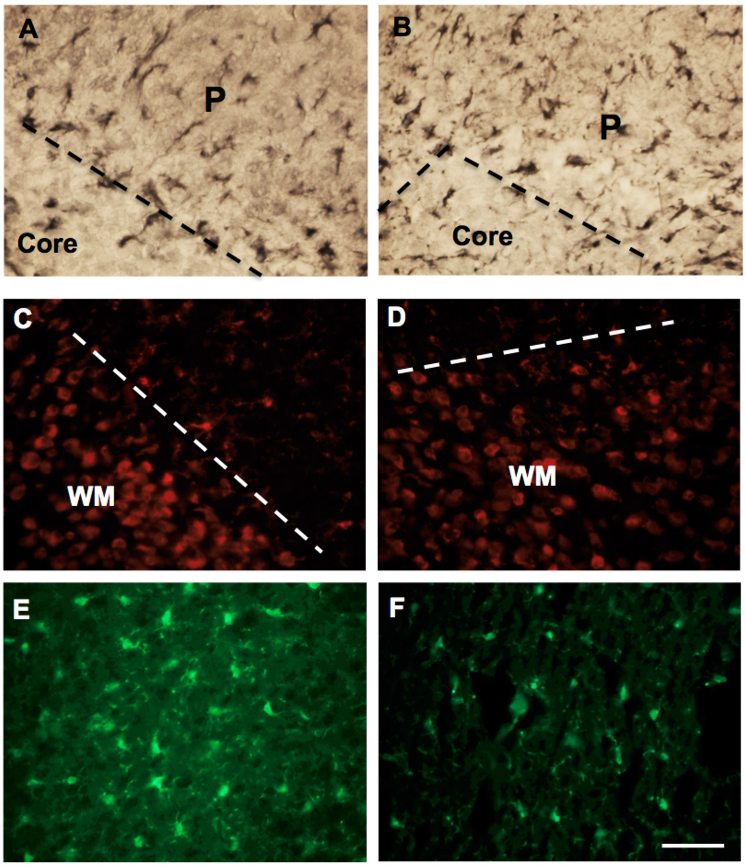

Figure 5: Immunohistochemistry for astrocyte (GFAP, A-B) and macrophage (tomato lectin, TL – C-D)/microglia (Iba-1, E-F) in PBS-treated animals (A, C, and E) and sildenafil 10 mg/kg

(B, D, and F) at 72 hours (A-D), and 8 days (E-F) after pMCAo in mouse pups. Scale bar represents 100 μm. P= penumbra; WM= white matter.

Supplemental Figure 6

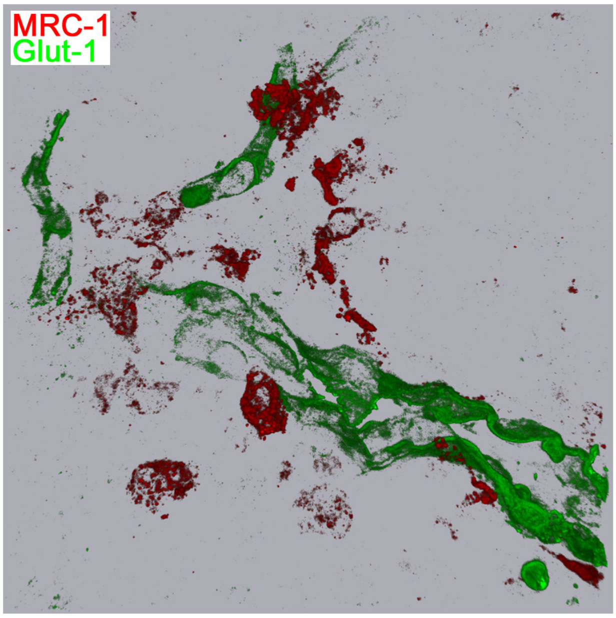

Figure 6: 3D confocal reconstruction of a microvessel stained with the 54 kDa Glut-1 protein (green) and microglia/macrophage stained with MRC-1 protein (M2-like marker).
