# Supplementary material for: Sildenafil, a cyclic GMP phosphodiesterase inhibitor, induces microglial modulation after focal ischemia in the neonatal mouse brain
Source: J Neuroinflammation. 2016 Apr 28;13:95. doi: 10.1186/s12974-016-0560-4 (PMC4850658; doi:10.1186/s12974-016-0560-4)
Supplement: Additional file 2: Table S1. — Primer sequences, protein targets, and NCBI references. (PDF 306 kb) [file 12974_2016_560_MOESM2_ESM.pdf]

Supplementary Table 1: Primer sequences, protein targets and NCBI references

Gene	Target protein and abbreviation	Sense	Anti-sense	NCBI Reference
GAPDH	Glyceraldehyde 3 phosphate dehydrogenase [illegible]	GGC CTT CCG TGT TCC TAC	TGT CAT CAT ATC TGG CAG GTT	NM_008084.2
CD32	Cluster of differentiation 32 (CD32)	CTG GAA GAA GCT GCC AAA AC	CCA ATG CCA AGG GAG ACT AA	NM_010187.2
CD86	Cluster of differentiation 86 (CD86)	GAG CGG GAT AGT AAC GCT GA	GGC TCT CAC TGC CTT CAC TC	NM_019388.3
Ptgs2	Cyclooxygenase-2 (Cox-2)	TCA TTC ACC AGA CAG ATT GCT	AAG CGT TTG CGG TAC TCA TT	NM_011198.3
CD206	Cluster of differentiation 206 (CD206)	CTT CGG GCC TTT GGA ATA AT	TAG AAG AGC CCT TGG GTT GA	NM_008625.2
Arg1	Arginase-1 (Arg1)	GTG AAG AAC CCA CGG TCT GT	GCC AGA GAT GCT TCC AAC TG	NM_007482.3
Lgals3	Galectin-3 (Gal-3)	GAT CAC AAT CAT GGG CAC AG	ATT GAA GCG GGG GTT AAA GT	NM_010705.3
Il1rn	Interleukin 1 receptor antagonist (IL-1Rn)	TTG TGC CAA GTC TGG AGA TG	TTC TCA GAG CGG ATG AAG GT	NM_031167.5
Il4ra	Interleukin 4 receptor alpha (IL-4Rα)	GGA TAA GCA GAC CCG AAG C	ACT CTG GAG AGA CTT GGT TGG	NM_001008700.3
